# Volitional EMG Control of a Novel Powered Ankle Prosthesis: A Case Series on Muscle Selectivity and Biomechanical Consequences

**DOI:** 10.3390/bioengineering13070722

**Published:** 2026-06-24

**Authors:** Faranak Rostamjoud, Mohamed Abdelbar, Friðrika Björk Þorkelsdóttir, Sophie Thiele, Anna Lára Ármannsdóttir, Atli Örn Sverrisson, Sigurður Brynjólfsson, Kristín Briem

**Affiliations:** 1 School of Health Sciences, University of Iceland, 102 Reykjavík, Iceland; kbriem@hi.is; 2 School of Engineering and Natural Sciences, University of Iceland, 102 Reykjavík, Iceland; msm18@hi.is (M.A.); sb@hi.is (S.B.); 3 Össur Ehf., Grjothals 1–5, 110 Reykjavík, Iceland

**Keywords:** co-contraction, gait analysis, residual-limb activation, transtibial amputation

## Abstract

This study investigated the feasibility and biomechanical effects of volitional electromyography (EMG)-based control of a powered transtibial ankle prosthesis. Four male participants completed static and dynamic EMG assessments and gait analysis while using both their prescribed passive prosthesis and an EMG-controlled powered prototype during level walking at self-selected and fast speeds, as well as ramp ascent and descent. Selective activation of residual tibialis anterior and gastrocnemius muscles was quantified using a co-contraction index, and lower-limb kinematics and kinetics were compared between prosthetic conditions. Participants were able to generate task-dependent residual muscle activity, supporting the feasibility of EMG-based volitional control. However, muscle selectivity was reduced during dynamic tasks, with higher co-contraction during gait than during seated static contractions, and substantial inter-subject variability was observed. Compared to the prescribed passive prosthesis, the EMG-controlled prototype generally produced lower prosthetic-side ankle range of motion and ankle power, although ankle moments were sometimes slightly greater. These findings suggest that EMG control is feasible, but that future controller design must remain flexible to individual users’ neuromuscular abilities and dynamic control limitations. The results provide important guidance for the development and testing of more adaptive, personalized, and functionally effective EMG-controlled prosthetic ankle systems.

## 1. Introduction

Lower-limb amputation significantly alters gait mechanics by disrupting the normal function of the ankle–foot complex. Transtibial amputees (TTAs) frequently experience restricted ankle motion and reduced push-off power, resulting in altered bilateral kinematics and kinetics during daily activities [1]. Conventional passive ankle–foot prostheses rely on energy storage–return mechanisms and are therefore unable to generate net positive mechanical power or actively adapt impedance in response to user intent. Due to the inherent limitations of such fixed, or manually adjustable properties, TTAs adopt compensatory strategies to maintain propulsion and stability, most notably increased reliance on proximal joints. These include elevated hip extensor and flexor moments on the prosthetic and, in many cases, the intact limb [2,3], and increased knee and hip flexion during swing to facilitate toe clearance [4]. Asymmetric gait patterns are also prevalent, characterized by longer stance time and higher loading rates on the intact limb [3,5]. Although these strategies support functional ambulation, they are associated with increased metabolic energy expenditure [6] and increased risk of secondary musculoskeletal conditions such as knee osteoarthritis [7,8].

Recent advances in powered and semi-active prosthetic ankle–foot systems have sought to address the limitations of passive devices [9]. Powered ankles can generate torque and positive work to improve push-off power and reduce the metabolic cost of walking [10,11]. Semi-active and variable-stiffness designs adjust forefoot stiffness or damping with relatively low energy consumption [12,13] to better accommodate changes in speed or terrain and potentially improve gait symmetry [14].

Within this broader trend, electromyography (EMG)-based or myoelectric control has emerged as a promising strategy to directly incorporate residual muscle activity into lower-limb prosthesis operation [15,16]. Despite muscle atrophy and altered anatomy, TTAs retain neuromuscular activity in residual limb muscles [17], and task-dependent EMG patterns can be recorded inside the prosthetic socket during gait [18]. A range of EMG-driven ankle control paradigms have emerged that vary in the extent to which EMG directly drives torque, modulates impedance, or informs state-based control [15]. Direct EMG-driven strategies may proportionally map residual muscle activity to prosthetic ankle torque, position, or impedance, enabling voluntary modulation [19,20], while remaining sensitive to signal variability and user effort. Hybrid EMG-based approaches, on the other hand, such as EMG-modulated impedance and finite state machine (FSM) controllers, embed muscle signals within predefined gait states or impedance profiles to adjust parameters like stiffness, damping, or push-off torque [21,22,23], improving intuitiveness while preserving stability.

Although studies have shown that TTAs can generate measurable and task-dependent EMG signals from residual muscles [17,24], most supporting data have been obtained under static or quasi-static conditions. Therefore, it remains unclear whether users can achieve selective and consistent muscle activation in dynamic situations where cognitive demands may induce greater antagonist muscle co-contraction or new aberrant gait patterns.

The practical adoption of EMG-based prosthetic control systems must consider the clinical value of improved biomechanical outcomes, within the context of the users’ ability to intuitively and consistently generate control signals during everyday activities without excessive effort. This is particularly important given the added complexity, cognitive demand, and engineering requirements associated with EMG-controlled powered prostheses.

Therefore, the primary aim of this study was to assess the feasibility of voluntary residual-limb EMG control by determining whether transtibial prosthesis users could generate measurable, selective task-dependent EMG signals during static and dynamic activities. As a secondary, exploratory analysis, lower-limb kinematics and kinetics were assessed across functional activities when participants used their prescribed passive prosthesis versus an EMG-controlled ankle that enabled volitional modulation of stiffness, damping, and push-off power. The integration of these analyses may elucidate whether EMG-based control provides an intuitive and functionally meaningful interface for prosthetic control, and inform the design of more effective user-driven prosthetic technologies.

## 2. Materials and Methods

### 2.1. Participants

Four male unilateral TTAs (mean ± SD age = 47 ± 10.2 years, weight = 90.2 ± 20.1 kg, height = 176.2 ± 8.4 cm) participated in the study. All were active community ambulators (Medicare Functional Classification Level (MFCL) K3 and K4) and routinely used a passive prosthetic ankle-foot device (Table 1). None of them had prior experience using a powered prosthetic ankle. Ethical approval was granted by the Icelandic National Bioethics Committee (protocol CII2025050655) and written informed consent was provided prior to data collection.

### 2.2. Static EMG Assessment

Prior to prosthetic fitting and familiarization with the EMG-controlled ankle system, participants attended a session to assess their ability to selectively activate residual limb muscles under controlled conditions, where visual real-time feedback was provided. During this session, participants were seated comfortably in a chair without wearing their prosthetic socket or liner. Surface EMG electrodes (Trigno™, Delsys Inc., Natick, MA, USA) were placed over the residual tibialis anterior (TA) and gastrocnemius (GAS) muscles, which were identified with ultrasound imaging [25]. Electrodes were then secured in place with elastic bandaging to maintain consistent skin contact throughout the session.

EMG signals were acquired using EMGworks Acquisition software V4.8.0 (Delsys Inc., Natick, MA, USA) at a sampling rate of 2148 Hz while using a second-order Butterworth band-pass filter (20–450 Hz), followed by computation of the root mean square (RMS) envelope with a 250 ms moving window.

The maximum voluntary contraction (MVC) value for each muscle was defined as the highest RMS value obtained from two 3 s MVC trials, in which participants imagined performing the corresponding ankle movements by activating the appropriate primary muscle (TA for dorsiflexion and GAS for plantarflexion) at maximum intensity without discomfort. Participants then performed 6 consecutive submaximal contractions for dorsiflexion and 6 for plantarflexion, aiming to reach approximately 40% of their MVC for the agonist while minimizing activation of the antagonist muscle. Each contraction was held for 3 s followed by a 10 s rest. A monitor provided participants with real-time visual feedback on agonist and antagonist muscle activity in the form of colored bars representing normalized EMG amplitude relative to MVC.

### 2.3. Dynamic EMG Assessment

Ultrasound imaging (Terason, Burlington, VT, USA) was used to locate the muscle bellies of TA and GAS [25] for appropriate electrode placement (two bipolar recording electrodes and one ground), confirm visible contractile activity, and verify that sufficient muscle tissue was available. The electrode placement was confirmed through signal quality checks, marked on the skin, and transferred to a prosthetic liner. For each participant, a customized EMG-enabled liner and socket was fabricated following the design described by Sverrisson and Sigurðardóttir [26].

During dynamic assessment, EMG signals were sampled at 1000 Hz while using a band-pass filter between 75 and 400 Hz, then full-wave rectified and low-pass filtered at 2 Hz to extract the linear envelope. An adaptive moving-average filter for artifact removal [26] was applied prior to use in the control algorithm. For each EMG channel, the mean of the most recent 10 samples was compared with the mean of the preceding 90 samples; when the short-term mean exceeded 1.7 times the preceding mean, the affected sample was replaced by the preceding mean. The MVC for each muscle was obtained while participants performed dorsiflexion and plantarflexion efforts for 3 s at maximal intensity without discomfort. Three repetitions were performed in a seated position and three in standing for each muscle. The highest amplitude obtained after signal filtering across all trials was defined as the MVC for each muscle. All EMG signals used for real-time prosthetic control and offline analyses were normalized to this MVC value within the same session. Trials were visually inspected for abrupt artifacts and signal loss. Trials with excessive contamination were repeated or excluded when necessary; no participant experienced persistent signal loss, and most trials were retained after filtering and quality checks.

#### 2.3.1. EMG-Based Prosthetic System

Participants were fitted with a prototype powered prosthetic ankle designed to operate in parallel with carbon-fiber blades of an Össur Pro-Flex Pivot foot, described in [27]. The system incorporated a powered ankle mechanism capable of generating up to 75 Nm of motor torque and was mechanically coupled to the blade through a custom double-link architecture, allowing adjustable stiffness, damping, and task-dependent push-off assistance.

An EMG-driven variable impedance controller (VIC) was used, where volitional user input is integrated with an FSM framework to modulate prosthesis behavior [23]. The four states of the FSM used in this study are controlled plantar flexion, controlled dorsiflexion, powered pushing, and swing. In each, the torque is expressed in Equation (1):

(1) $$\tau_{FSM}=k_{n}\left({\theta_{n}^{*}-\theta }_{a}\right)-b_{n}\dot{\theta_{a}}$$

where the ankle angle and angular velocity are represented by $\theta_{a}$ and $\dot{\theta_{a}}$, while $k_{n}$, $b_{n}$, and $\theta_{n}^{*}$ represent the stiffness coefficient, damping coefficient, and equilibrium point, respectively, of the impedance controller of each state [28,29]. The VIC continuously adjusts the $k_{n}$ in the impedance control rule in Equation (1) for the controlled dorsiflexion and powered pushing subphases over time, in response to EMG data from the residual muscles and the ankle angle [23]. During the swing phase, the controller behavior was represented as position control of the ankle joint [30]. The stiffness value was kept constant throughout the controlled plantar flexion subphase to prevent excessive variability at heel strike. During ramp descent, the powered pushing subphase was disabled for safety [31]. In addition, the damping coefficient $b_{n}$ is continuously adjusted in the impedance control rule in Equation (1) for the controlled dorsiflexion subphase based on both EMG signals from the residual muscles and the ankle angle.

#### 2.3.2. Training and Familiarization

Participants completed 5–7 training sessions prior to data collection with each session lasting approximately 2 h. All participants completed at least 10 h of practice, with additional training provided when needed based on performance and comfort. Training initially focused on familiarization with the powered ankle without implementing EMG control, allowing them to adapt to the mechanical characteristics of the powered ankle system. Subsequently, several training sessions introduced voluntary EMG-based control.

During EMG-based control training, participants were taught how to use residual muscle activation to modulate prosthesis behavior across different tasks. For level-ground walking and ramp ascent, participants were encouraged to voluntarily activate the GAS during late stance to enhance push-off power. For ramp descent, since the powered push-off function was disabled, participants were instructed to use muscle activation, preferably the TA, to modulate ankle damping during stance, allowing them to better control forward progression. Control parameters were tuned individually based on user performance and preference during these sessions. These included the dorsiflexion threshold for initiating the powered-pushing phase, level of push-off power and the minimum and maximum stiffness and damping values for each gait task.

#### 2.3.3. Gait Analysis

A within-subject experimental design was used to compare participants’ gait parameters with their prescribed passive prosthesis and the EMG-controlled active prosthesis. Biomechanical data were collected while participants performed level-ground walking (LGW) at self-selected (SSS) and fast speeds (FS), as well as during ramp ascent and descent (7.5° incline) at their SSS. The order of prosthetic conditions was counterbalanced across participants, with two participants beginning with the prescribed passive prosthesis and two beginning with the EMG-controlled prototype.

Three-dimensional kinematics and ground-reaction forces were recorded using a 10-camera motion capture system (Vicon Motion Systems, Vicon, Oxford, UK) synchronized with six force plates (AMTI, Watertown, MA, USA). Marker trajectories were sampled at 100 Hz, and force data at 1000 Hz. A customized lower-body marker set was used to construct a cluster-based six-degree-of-freedom (6DOF) biomechanical model. Markers were placed bilaterally on anatomical landmarks of the pelvis, thigh, shank, and foot, including the anterior and posterior superior iliac spine, medial and lateral femoral epicondyles, medial and lateral malleoli, the calcaneus, and the first and fifth metatarsals. Each body segment (pelvis, thigh, shank, and foot) was additionally tracked with a rigid 4-marker cluster. Static calibration trials were recorded with all anatomical markers in place, after which only tracking clusters and required segment-defining markers were retained for dynamic trials. Marker trajectories were filtered using a 4th-order zero-lag low-pass Butterworth filter with a cutoff frequency of 6 Hz. Force-plate signals were filtered using a 2nd-order zero-lag low-pass Butterworth filter with a cutoff frequency of 50 Hz.

Kinematic and kinetic data were processed using Vicon Nexus version 2.19 (Vicon Motion Systems, Vicon, Oxford, UK) and Cleanse version 2026.1.0 (Moveck Solution Inc., Quebec City, QC, Canada) software. Further analyses were performed using MATLAB version 23.2 (MathWorks, Natick, MA, USA). All trials were visually inspected for marker loss and data quality. Kinetic data were normalized by body mass.

### 2.4. Data Processing and Analysis

EMG data collected during the static condition were further reprocessed offline using a second-order Butterworth high-pass filter at 25 Hz and a band-stop filter with cutoff frequencies of 49–51 Hz to remove powerline interference. The RMS of the filtered signal was then calculated for subsequent analysis.

To evaluate selective muscle activation under static and dynamic conditions, a co-contraction index (CCI) was computed to quantify the relative activation of antagonist muscles during each task. The CCI was defined as:

(2) $$CCI=\frac{{EMG}_{non\text{-}primary}}{{EMG}_{primary}}\;\times \;100$$

where *EMG_primary_* corresponds to the normalized RMS amplitude of the agonist and *EMG_non-primary_* represents the antagonist muscle during each task. Higher CCI values indicate greater muscle co-contraction (reduced selectivity of muscle activation). The mean and SD for each participant’s CCI was calculated for both static and dynamic conditions. In static condition, the CCI was computed for each of the six repetitions per movement, and during dynamic activities (walking and ramp tasks), the CCI was calculated over multiple gait cycles.

Normality assumptions were not met for the EMG-derived outcome measures; therefore, non-parametric analyses were applied. For each participant separately, Kruskal–Wallis tests were used to assess differences in maximum normalized muscle amplitude and maximum CCI across activities. Significant main effects were followed by pairwise Wilcoxon rank-sum tests with Holm correction for multiple comparisons.

Joint kinematics and kinetics for the ankle, knee, and hip were extracted bilaterally for each task. EMG-controlled and passive prosthesis conditions were compared using linear mixed-effects models (LMM). Models were fitted separately for joint range of motion (ROM), joint moments, and joint power. Side (prosthetic, intact), prosthetic foot (EMG-based vs. prescribed) and activity (LGW-SSS, LGW-FS, ramp ascent and descent) were entered as fixed effects together with all interaction terms, and subject-specific variability was accounted for by including participant ID as a random intercept. Normality was evaluated by visual inspection of Q–Q plots of the residuals. Type III ANOVA was used on the constructed models to test the significance of fixed effects. When interactions or main effects were significant, post hoc pairwise comparisons of estimated marginal means were performed. Statistical significance was set at *p* < 0.05.

## 3. Results

**Figure 1:**
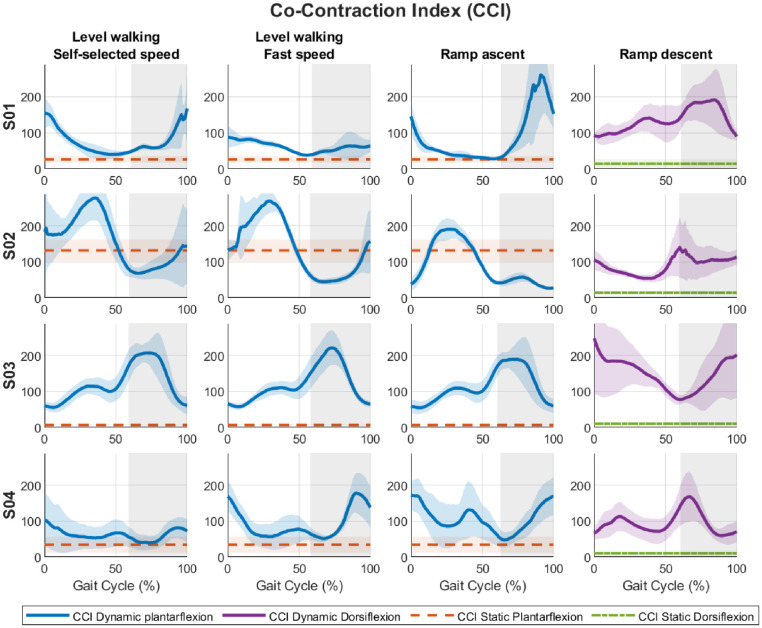
Co-contraction index (CCI) across the gait cycle for each participant (S01–S04) during level-ground walking at self-selected and fast speeds, ramp ascent, and ramp descent. The gray background denotes the swing phase.

Co-contraction during gait generally exceeded the co-contraction observed during the seated static task, indicating reduced selectivity of residual muscle activation under dynamic conditions (Figure 1). This pattern was also evident when CCI was evaluated specifically during the gait phases in which active EMG control was enabled (Table 2). During the powered pushing phase defined from the prosthetic foot state, mean dynamic CCI ranged from 47.3 to 174.6 in LGW-SSS, 45.3 to 151.3 in LGW-FS, and 32.2 to 127.4 during ramp ascent. During ramp descent, where active EMG control was applied throughout stance, mean dynamic CCI ranged from 77.3 to 142.9. Maximum dynamic CCI throughout the gait cycle showed activity-dependent differences in S01, S02, and S04 (*p* = 0.0004, *p* = 0.0035, and *p* = 0.0087, respectively), but not in S03 (*p* = 0.1384).

**Figure 2:**
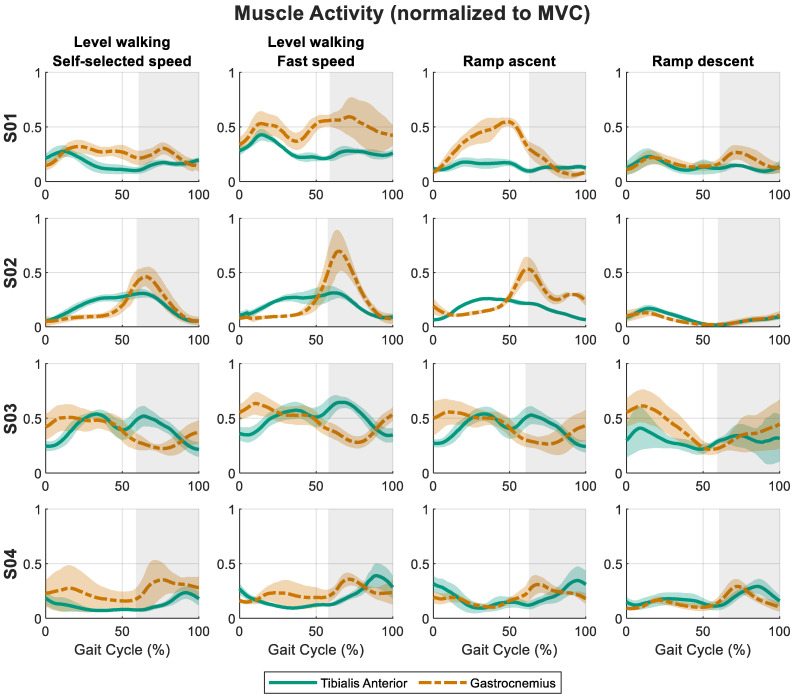
Normalized residual-limb muscle activity across the gait cycle for each participant (S01–S04) during level-ground walking at self-selected and fast speeds, ramp ascent, and ramp descent. The gray background denotes the swing phase.

During the dynamic gait tasks, residual-limb muscle activity varied across activities and between participants (Figure 2). Maximum normalized GAS amplitude showed activity-dependent differences in S01, S02, and S04 (all *p* < 0.02, Figure 2), but not in S03 (*p* = 0.432). Post hoc comparisons confirmed significant differences in ramp ascent vs. both LGW-SSS and ramp descent in S01 (*p* = 0.005 and 0.004), LGW-SSS vs. ramp descent in S02 (*p* = 0.020), and LGW-SSS vs. ramp descent in S04 (*p* = 0.033). Maximum normalized TA amplitude varied by activity in all participants (all *p* < 0.002, Figure 2), although significant post hoc differences were limited to LGW-SSS vs. ramp descent in S02 (*p* = 0.020), LGW-FS vs. ramp ascent and LGW-SSS in S03 (*p* = 0.003 and 0.004), and ramp ascent vs. LGW-SSS in S04 (*p* = 0.024).

Joint kinematics and kinetics differed between the prescribed passive prosthesis and the EMG-controlled prototype prosthesis across walking conditions (Appendix A). On the prosthetic side, there was a significant main effect of prosthetic foot on ankle ROM (*p* < 0.0001), moment (*p* = 0.005), and power (*p* < 0.0001). Post hoc comparisons showed that ROM and power were significantly greater with the prescribed foot than with the EMG-based foot across all activities (all *p* < 0.0001). In contrast, ankle moment was slightly greater with the EMG-based foot than with the prescribed foot across activities, although this difference was statistically significant only during LGW-SSS (*p* = 0.0002). Prosthetic-side knee and hip variables showed less consistent participant- and task-specific changes. On the intact side, condition effects were smaller and less uniform, with some reductions in ankle ROM and power, particularly during level walking and ramp descent, but otherwise mixed changes at the ankle, knee, and hip.

## 4. Discussion

This study examined whether residual-limb EMG could be used as a practical control input for an active transtibial prosthesis during functional gait tasks, and whether such control altered lower-limb biomechanics relative to each participant’s prescribed passive prosthesis. The primary success criterion in this feasibility case series was the ability of participants to voluntarily generate measurable, task-dependent residual-limb EMG signals during functional activities. Co-contraction was used to characterize signal selectivity, whereas the biomechanical measures were secondary outcomes describing the consequences of the current prototype. Overall, the findings show that participants were able to generate task-dependent EMG signals from the residual TA and GAS muscles, supporting the feasibility of volitional EMG input during walking. At the same time, the results also show substantial inter-subject variability, reduced activation selectivity during dynamic tasks compared with static seated contractions, and generally lower ankle ROM and ankle power with the EMG-controlled prototype than with the prescribed passive prosthesis. Together, these findings suggest that voluntary EMG control is feasible and was supported by the prototype’s ability to adapt actuator output across tasks, with greater assistance generated during ramp ascent than during level-ground walking through EMG- and ankle-angle-based modulation. However, its effective integration into gait remains limited by signal selectivity, timing, task complexity, and the present state of controller tuning and hardware implementation.

The main finding of this study is that residual-limb muscle activity during gait was neither absent nor random. Rather, TA and GAS activation varied with walking conditions, supporting the basic premise of EMG-based ankle control in transtibial prosthesis users. At the same time, the EMG profiles showed considerable variability between participants, indicating that the ability to generate and use these signals was highly individualized. Despite this inter-subject variability, a qualitative consistency was preserved within participants across tasks. Each participant tended to use a similar overall muscle-activation strategy across level walking, fast walking, and ramp conditions, while contraction amplitude varied according to the functional demands of the task. This may reflect a user-specific motor strategy that is retained across activities once the user has learned to interact with the controller.

Another important finding is that co-contraction during gait generally exceeded that observed in the seated static condition. In the seated task, participants activated residual muscles under relatively simple conditions, without the need to balance, walk, or coordinate whole-body movement, and with the benefit of direct visual feedback to support selective recruitment. During dynamic tasks, however, they had to simultaneously regulate progression, stability, timing, and prosthesis control, likely increasing the cognitive and neuromotor demands of the task. The higher co-contraction observed during gait therefore likely reflects the greater neuromotor complexity of dynamic ambulation and indicates that success in a static EMG test does not necessarily translate to equally selective control during walking. This discrepancy may also reflect a training gap rather than a fixed physiological limitation. Progressive rehabilitation strategies may help users transfer selective residual-muscle recruitment from low-demand conditions into functional tasks, for example through real-time EMG biofeedback that progresses from seated or supported practice to weight-bearing and gait-specific training [32,33]. Motor imagery or virtual EMG practice may also be useful as an adjunct [34], although evidence for improving lower-limb prosthetic EMG control specifically remains limited. This difference may also be partly methodological, as the static and dynamic EMG recordings used different electrode setups, electrode sizes/placement conditions, and signal-processing pipelines; moreover, socket-based recordings during ambulation are more susceptible to movement artifacts, soft-tissue motion, and pressure-related signal changes. Some other factors may contribute to increased co-contraction during gait, including difficulty isolating residual muscles within the socket, the need for limb stiffening to enhance perceived stability, uncertainty about activation timing, and the cognitive demand of coordinating muscle activity with gait. From a control perspective, such co-contraction may reduce the clarity of the EMG command signal, particularly in systems that rely on differential activation of antagonist muscle groups. However, if a user’s activation pattern remains stable within and across tasks, it may still provide a usable control signal at key phases of gait, such as for modulating stance damping or contributing to push-off. Accordingly, the challenge for future systems may not be simply to eliminate co-contraction, but to identify when consistent user-specific activation patterns can still be harnessed effectively. Future studies should complement CCI with quantitative measures of phase-specific timing, within-user repeatability, and separability between intended control states.

During tasks expected to require greater ankle contribution, such as ramp ascent or faster walking, some participants showed modestly greater activation amplitudes, which would be consistent with a need for greater prosthetic assistance or stronger controller modulation. Ramp descent, however, did not produce a clear shift toward a distinct TA-dominant strategy in all participants, despite TA being the suggested muscle for stance damping in this condition. Instead, some participants appeared to retain a contraction pattern similar to that used in other tasks, whereas others showed relatively limited activity. This suggests that reconfiguring muscle recruitment for task-specific control during dynamic locomotion may be difficult, even after training, especially when control demands are continuous and time-constrained. Future systems may therefore benefit from adapting to each user’s habitual activation pattern, rather than requiring a task-specific idealized pattern with clear TA–GAS separation across all conditions.

The results indicate that not only EMG magnitude but also EMG timing varied across tasks. This is particularly important in a gait-phase-dependent controller, where the timing of a muscle burst may be as important as its amplitude. If the intended contraction occurs too late in stance, the controller may deliver stiffness, damping, or push-off assistance after the biomechanically optimal period. In that case, users may still generate a meaningful volitional command, but the prosthesis may fail to convert that command into functionally effective propulsion or stance control. This interpretation is consistent with the present findings: in some cases, peak muscle activity occurred when the foot had already entered swing, while the EMG-controlled ankle generally showed lower prosthetic-side ankle power than the passive condition. Together, these results suggest that part of the intended assistance may have been delivered too late relative to toe-off, shifting the power-generation profile later than desired and reducing its contribution during the most functionally beneficial portion of stance. However, the temporal mismatch between muscle activation and prosthetic response was not quantified using a formal latency analysis. Therefore, this interpretation should be considered preliminary. Future studies should use synchronized EMG, controller-state, motor-command, and prosthetic-motion data to quantify end-to-end latency and distinguish delays associated with signal processing, controller execution, and mechanical response. The absence of continuous visual EMG feedback during walking practice may also have limited participants’ ability to learn the timing and magnitude of activation required for effective push-off and damping modulation. Future improvements may therefore require not only better user training, but also better synchronization between user intent and prosthetic response. Approaches such as increasing the low-pass filter cutoff frequency, earlier detection thresholds, user-specific phase-dependent gains, or timing-compensation strategies may help align EMG-driven assistance more effectively with the gait cycle, although this must be balanced against increased susceptibility to noise and motion artifacts.

The combination of reduced ROM with increased ankle moment but lower ankle power observed in our biomechanical results suggests that the prosthetic ankle may have been generating torque without converting it into fast, well-timed ankle motion. Because joint power depends on both joint moment and angular velocity, a smaller or slower plantarflexion excursion could reduce push-off power even when ankle moment is maintained or increased. This may indicate that the controller produced a relatively stiff or resistive ankle response, in which support moments are present but the timing and velocity of motion are insufficient to generate strong push-off power. Therefore, future development should focus on tuning the relationship between voluntary EMG activation and the intended biomechanical response, so that residual-muscle input not only triggers or modulates prosthesis behavior, but produces appropriately timed and task-relevant changes in ankle motion, stiffness, damping, or push-off assistance.

The biomechanical findings do not support a single global conclusion that the EMG-controlled prosthesis reduced sagittal-plane compensatory joint demands relative to the prescribed passive prosthesis. Instead, compensation appeared to remain user-specific and was distributed across joints and tasks. S01 showed an overall reduction in sagittal-plane output on the prosthetic side with the EMG-controlled prosthesis across gait tasks. During level walking, prosthetic ankle ROM and power both decreased, as did knee moment and power, while hip changes were small or inconsistent. The same pattern was observed during ramp descent and ascent: ankle power dropped substantially, while neither the knee nor the hip showed a strong, repeated increase that would support compensation primarily through one proximal joint. This agrees with the broader literature showing that reduced distal function does not always produce a single larger proximal moment, but may instead lead to more diffuse gait adaptations [1,35]. In contrast, S02 showed distribution toward the prosthetic knee in both level-ground conditions with the EMG-controlled prosthesis, where ankle ROM and power were markedly lower, but knee ROM and power increased. At the same time, hip ROM, moment, and often power, decreased, and so the pattern is more consistent with knee-dominant than hip-dominant compensation. During ramp ascent, S02 appeared to derive the clearest functional benefit from the EMG-based ankle with greater ankle power, knee ROM and moment, and similar or slightly increased hip power. This is consistent with prior work showing that improved ankle push-off can reduce some compensations, particularly during uphill walking, without fully eliminating proximal adaptations [36]. S03 showed persistent distal limitation with the EMG-controlled prosthesis, without a clear sagittal-plane compensatory pattern. In level walking, kinematic and kinetic parameters were generally lowered and the same trend was observed during ramp descent. Ramp ascent differed slightly in that knee ROM increased, but knee moment and power still decreased and ankle power remained reduced. For this reason, S03 cannot be confidently characterized as demonstrating either knee-based or hip-based compensation. Instead, the pattern may reflect compensations not captured by the peak sagittal metrics analyzed here, including contralateral loading, trunk or pelvic adjustments, transverse- or coronal-plane mechanics, or temporal asymmetry [37,38]. In S04, ankle ROM decreased consistently with the EMG-controlled prosthesis across tasks, whereas ankle moment often increased and ankle power remained lower. During level walking, knee power increased while knee moment was slightly lower or unchanged, suggesting some redistribution toward the prosthetic knee. During ramp descent and ascent, however, this knee-based pattern was less consistent, and the overall response again appeared more mixed.

Although no participant showed signs of muscle cramps or discomfort during data collection and rest was provided as needed, muscle fatigue should still be considered as a possible contributor to the observed variability in dynamic EMG performance. Repeated walking trials and repeated efforts to produce selective contractions could have affected the consistency, amplitude, or selectivity of residual muscle activation over time, even if contraction levels during dynamic tasks did not appear to exceed roughly half of maximal effort. Residual-limb muscles may fatigue differently from intact-limb muscles because of altered anatomy, socket pressures, and the unusual demand of producing deliberate control signals during gait. Therefore, future studies would benefit from explicitly monitoring fatigue, for example by testing changes in EMG features over time, perceived exertion, or pre/post selectivity tests.

An important practical observation from this study is that users’ preferences differed in terms of prosthetic control settings. The level of push-off power and the range of stiffness and damping modulation were tuned according to those preferences during training. The preferred assistance and resulting actuator behavior differed between participants and across tasks. None of the participants preferred maximum push-off assistance during level-ground walking; however, during the more demanding ramp-ascent task, actuator output increased and could reach the maximum available torque. This task-dependent adaptation was achieved through modulation based on both residual-limb EMG signals and ankle angle. This suggests that future systems should prioritize personalized, task-dependent modulation of assistance, controllability, and comfort rather than consistently maximizing motor output. Participants’ verbal feedback further suggested that EMG control may be most acceptable when it is available selectively for more demanding tasks, layered on top of a reliable autonomous baseline controller, rather than as a continuously active control mode during all steps.

Several additional limitations should be acknowledged. First, the sample size was small, which limits statistical power and the generalizability of the findings. The participants were all male, unilateral transtibial amputees, and relatively high-functioning ambulators, so the results may not extend to broader prosthesis-user populations. Second, although participants completed familiarization and training, this exposure was still limited and may not have been sufficient for full adaptation to the system. Third, the study focused primarily on EMG behavior and sagittal-plane biomechanical outcomes, and did not directly quantify factors such as cognitive load, fatigue, metabolic cost, user effort, or balance confidence, all of which are likely relevant to the practical value of this control approach. Although ultrasound confirmed identifiable and contractile TA and GAS muscle tissue in all participants, residual-limb length, muscle volume, soft-tissue coverage and detailed surgical history were not systematically quantified. These anatomical factors may have contributed to the observed inter-subject variability in EMG quality and control performance. Participants also may not consistently reproduce a true maximal contraction. An over- or underestimated MVC could affect normalized EMG amplitudes and, because TA and GAS were normalized to separate MVC values, could bias the CCI in either direction. An additional limitation is that residual-limb EMG was recorded within the prosthetic interface, where signal quality may be influenced by electrode placement consistency, soft-tissue movement, socket pressure, perspiration, and motion artifact. Although signal quality was checked before testing and monitored continuously, the gradual changes cannot be fully excluded. As a result, some of the observed variability and reduced selectivity during gait may reflect not only user control limitations, but also limitations of the recording interface under dynamic conditions. In addition, Because the prescribed passive prosthesis and the EMG-controlled prototype differed not only in control strategy but also in hardware, foot geometry, alignment, build height, familiarity, and other mechanical characteristics, the observed biomechanical differences cannot be attributed specifically to EMG modulation. A powered-prosthesis condition using an autonomous or default controller would have helped isolate the effect of EMG input. Accordingly, the reductions in ankle ROM and power should be interpreted as effects of the overall prototype condition, rather than as effects attributable specifically to EMG modulation, because they may reflect the combined influence of stiffness, damping, alignment, foot geometry, and controller behavior. In two participants, the EMG-controlled prosthetic side had to be tested without a shoe because the longer residual-limb/prosthesis build height and ankle setting prevented shoe use, whereas the other two participants wore shoes bilaterally in both prosthetic conditions. These differences may have affected ankle–foot mechanics and should be considered when interpreting condition-related changes in ROM, moments, and power. Finally, the current findings also highlight limitations of the prototype ankle and its control framework. Although the system allowed volitional modulation of stiffness, damping, and push-off within a finite-state controller, the resulting gait mechanics did not surpass the prescribed passive prosthesis in the main ankle variables reported here. This suggests that the present implementation did not yet convert user input into biomechanical benefit in a robust way. Because the prototype was under development, the present results should be interpreted as identifying design constraints and opportunities for refinement rather than as a definitive test of the clinical value of EMG-controlled ankle prostheses.

## 5. Conclusions

In conclusion, this study shows that transtibial prosthesis users can voluntarily generate residual-limb EMG signals during walking and ramps tasks, supporting the feasibility of EMG-based volitional input for powered ankle prosthesis control. However, the practical use of those signals for prosthetic control remains constrained by limited selectivity in dynamic tasks, inter-subject variability, and a potential mismatch between timing of muscle activation and prosthetic response. Compared with the prescribed passive prosthesis, the present EMG-controlled prototype generally reduced prosthetic-side ankle ROM and ankle power, indicating that the current implementation did not yet restore more effective ankle function. Nevertheless, the participant-specific EMG patterns, the preserved ability to modulate muscle activity across tasks, and the positive user perception of EMG control for more demanding activities all suggest that EMG-based control remains promising. Therefore, the findings should be interpreted as evidence of feasibility, identifying design constraints and opportunities for refinement rather than as a definitive test of the clinical value of EMG-controlled ankle prostheses. The present findings provide useful guidance for improving timing of muscle activation for effective control, personalization, signal interpretation, and task-dependent deployment in future EMG-controlled prosthetic ankle systems.

## Figures and Tables

**Table 1:** Participant characteristics.

ID	Age (Years)	Weight (kg)	Height (cm)	Post Amputation (Years)	Cause of Amputation	Prescribed Foot
S01	58	112	179	20	Trauma	Pro-Flex Terra (Össur, Reykjavik, Iceland)
S02	51	66	165	51	Congenital	Pro-Flex XC (Össur, Reykjavik, Iceland)
S03	34	99	176	27	Neurofibromatosis	Pro-Flex XC (Össur, Reykjavik, Iceland)
S04	45	84	185	28	Trauma	Pro-Flex XC (Össur, Reykjavik, Iceland)

**Table 2:** Mean ± SD co-contraction index (CCI) during the gait phases in which active EMG control was enabled, together with the corresponding static CCI. Dynamic CCI was calculated during the powered pushing phase for level-ground walking at self-selected speed (LGW-SSS), level-ground walking at fast speed (LGW-FS), and ramp ascent, and during the stance phase for ramp descent.

ID	Dynamic CCI				Static CCI	
	LGW_SSS (Powered Pushing)	LGW_FS (Powered Pushing)	Ramp Ascent (Powered Pushing)	Ramp Descent (Stance)	Plantarflexion	Dorsiflexion
S01	47.3 ± 7.9	45.3 ± 5.8	32.2 ± 3.5	93.3 ± 13.8	24.9 ± 7.4	12.9 ± 3.4
S02	174.6 ± 80.4	151.3 ± 84.0	127.3 ± 57.6	142.9 ± 32.0	131.8 ± 30.3	14.2 ± 4.7
S03	140.5 ± 39.7	146.1 ± 40.0	121.0 ± 29.9	77.3 ± 18.9	7.0 ± 1.2	10.1 ± 2.8
S04	55.6 ± 8.6	66.2 ± 9.0	91.5 ± 23.8	130.9 ± 23.5	34.2 ± 22.2	10.6 ± 7.2

**Figure A1:**
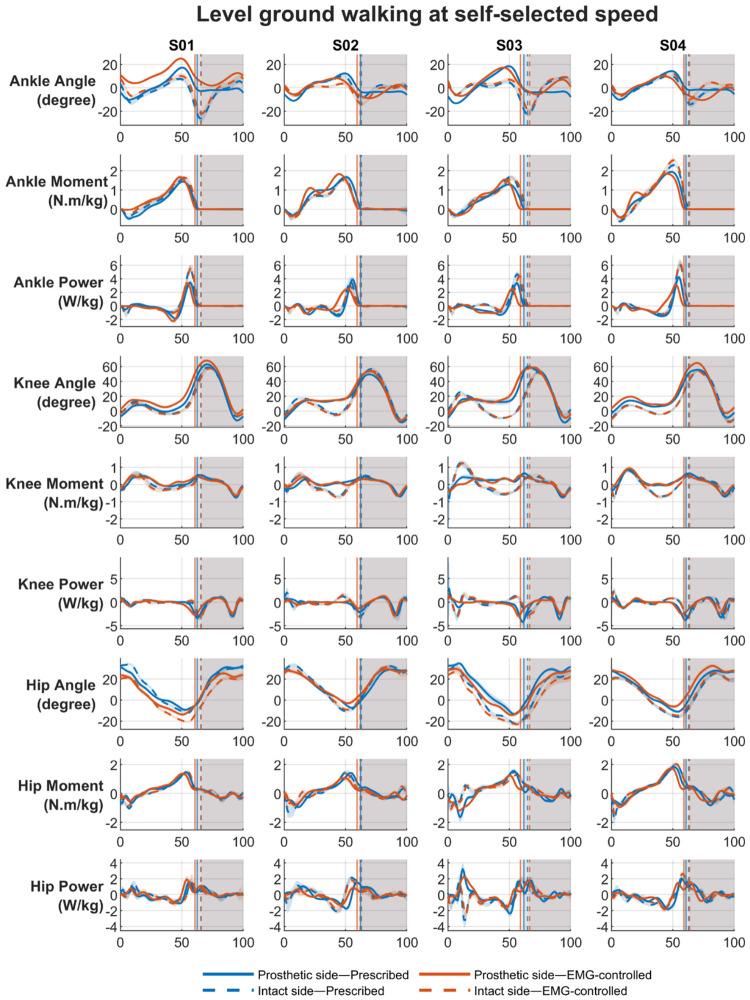
Mean sagittal-plane joint angle, moment and power profiles of the ankle, knee, and hip across the gait cycle for the prosthetic and intact limbs during level-ground walking at self-selected speed, comparing the prescribed passive prosthesis and the EMG-based ankle prototype. The gray background indicates the swing phase. Vertical lines denote the toe-off event for each condition.

**Figure A2:**
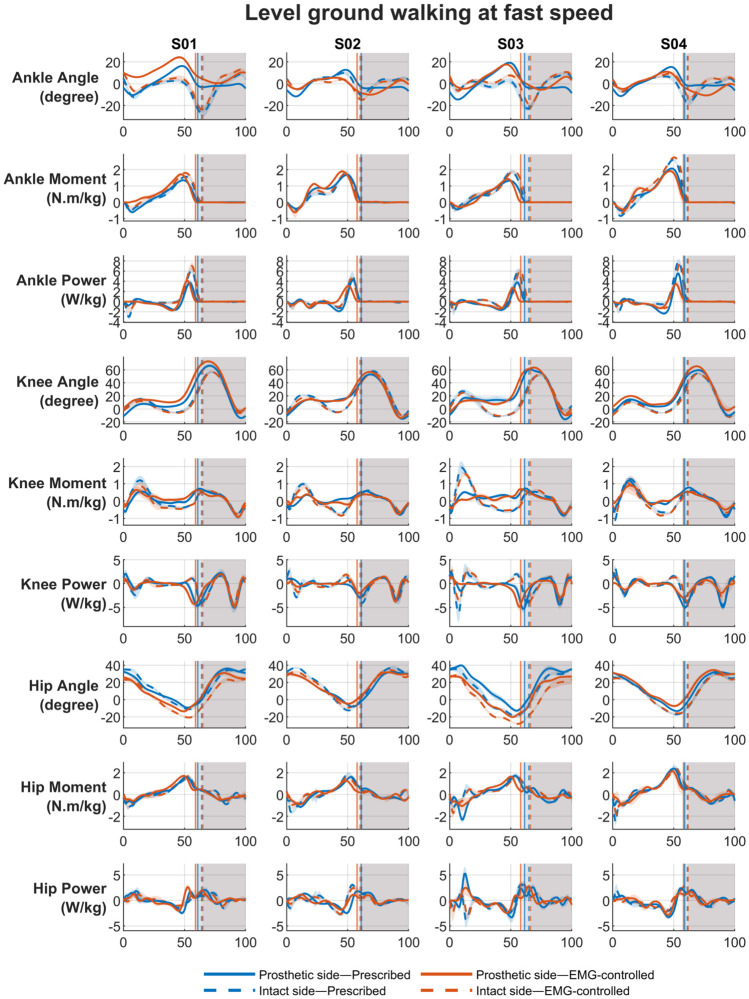
Mean sagittal-plane joint angle, moment and power profiles of the ankle, knee, and hip across the gait cycle for the prosthetic and intact limbs during level-ground walking at fast speed, comparing the prescribed passive prosthesis and the EMG-based ankle prototype. The gray background indicates the swing phase. Vertical lines denote the toe-off event for each condition.

**Figure A3:**
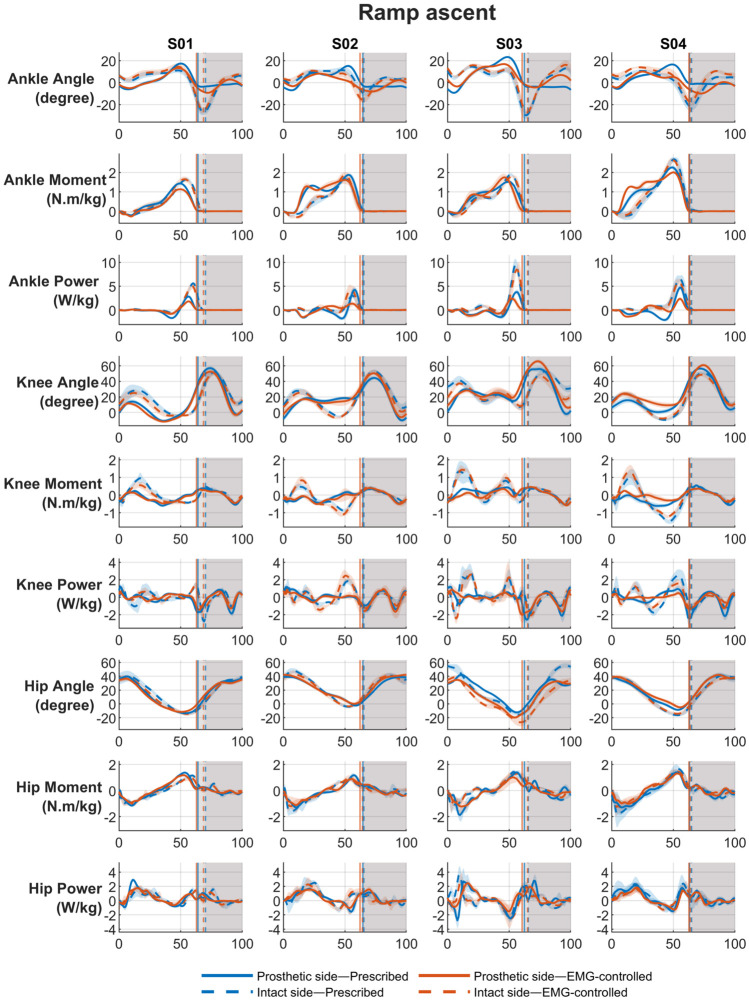
Mean sagittal-plane joint angle, moment and power profiles of the ankle, knee, and hip across the gait cycle for the prosthetic and intact limbs during ramp ascent, comparing the prescribed passive prosthesis and the EMG-based ankle prototype. The gray background indicates the swing phase. Vertical lines denote the toe-off event for each condition.

**Figure A4:**
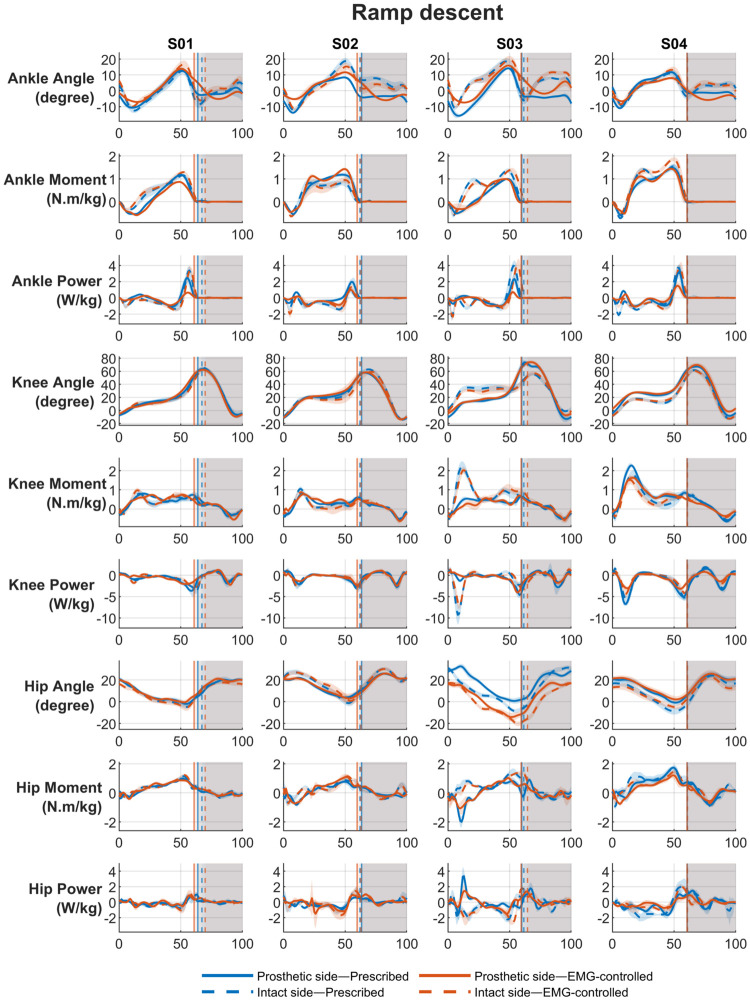
Mean sagittal-plane joint angle, moment and power profiles of the ankle, knee, and hip across the gait cycle for the prosthetic and intact limbs during ramp descent, comparing the prescribed passive prosthesis and the EMG-based ankle prototype. The gray background indicates the swing phase. Vertical lines denote the toe-off event for each condition.

## Data Availability

The data that supports the findings of this study are available from the authors upon reasonable request.

## Abbreviations

The following abbreviations are used in this manuscript:

EMG	Electromyography
TTA	Transtibial amputee
FSM	Finite state machine
MFCL	Medicare Functional Classification Level
TA	Tibialis anterior
GAS	Gastrocnemius
RMS	Root mean square
MVC	Maximum voluntary contraction
VIC	Variable impedance controller
LGW	Level-ground walking
SSS	Self-selected
FS	Fast speed
ROM	Range of motion
CCI	Co-contraction index
LMM	linear mixed-effects model
